# Comparing broad and narrow phenotype algorithms: differences in performance characteristics and immortal time incurred

**DOI:** 10.3389/jpps.2023.12095

**Published:** 2024-01-03

**Authors:** Joel N. Swerdel, Mitchell M. Conover

**Affiliations:** ^1^ Observational Health Data Analytics, Global Epidemiology, Janssen Research and Development, Titusville, NJ, United States; ^2^ Observational Health Data Sciences and Informatics, New York, NY, United States

**Keywords:** phenotype algorithms, immortal time, validation, observational data, bias

## Abstract

**Introduction:** When developing phenotype algorithms for observational research, there is usually a trade-off between definitions that are sensitive or specific. The objective of this study was to estimate the performance characteristics of phenotype algorithms designed for increasing specificity and to estimate the immortal time associated with each algorithm.

**Materials and methods:** We examined algorithms for 11 chronic health conditions. The analyses were from data from five databases. For each health condition, we created five algorithms to examine performance (sensitivity and positive predictive value (PPV)) differences: one broad algorithm using a single code for the health condition and four narrow algorithms where a second diagnosis code was required 1–30 days, 1–90 days, 1–365 days, or 1- all days in a subject’s continuous observation period after the first code. We also examined the proportion of immortal time relative to time-at-risk (TAR) for four outcomes. The TAR’s were: 0–30 days after the first condition occurrence (the index date), 0–90 days post-index, 0–365 days post-index, and 0–1,095 days post-index. Performance of algorithms for chronic health conditions was estimated using PheValuator (V2.1.4) from the OHDSI toolstack. Immortal time was calculated as the time from the index date until the first of the following: 1) the outcome; 2) the end of the outcome TAR; 3) the occurrence of the second code for the chronic health condition.

**Results:** In the first analysis, the narrow phenotype algorithms, i.e., those requiring a second condition code, produced higher estimates for PPV and lower estimates for sensitivity compared to the single code algorithm. In all conditions, increasing the time to the required second code increased the sensitivity of the algorithm. In the second analysis, the amount of immortal time increased as the window used to identify the second diagnosis code increased. The proportion of TAR that was immortal was highest in the 30 days TAR analyses compared to the 1,095 days TAR analyses.

**Conclusion:** Attempting to increase the specificity of a health condition algorithm by adding a second code is a potentially valid approach to increase specificity, albeit at the cost of incurring immortal time.

## Introduction

Phenotype algorithms are often the first component needed to conduct observational research studies. When developing these algorithms, there is usually a trade-off between definitions that are broad, prioritizing a lower number of false negative subjects, and narrow, prioritizing a lower number of false positive subjects. Narrow phenotypes for health conditions ensure that the cohorts selected by the algorithm are more likely to have the health conditions of interest.

A common method for developing narrow phenotypes and thereby increasing the specificity of an algorithm for a health condition is to require a second occurrence of the condition after the initial occurrence, which is the defined date of cohort entry. The required timing of the second code relative to the first is often inconsistent across studies. There are many examples of using this approach. For example, a literature search for algorithms for atrial fibrillation produced 39 research studies using computable phenotype algorithms. Of these, 16 used algorithms that required two or more diagnosis codes for atrial fibrillation. While requiring a second diagnosis code is a common practice, the time between the first and second codes varies widely. For example, O’Neal et al. required a second code between 7 days and 1 year after the first code [1]. Wilson et al. required a second code any time within 1 year from the initial diagnosis code [2]. Willey et al. required two diagnosis codes within the 3 years of their study period [3].

Algorithms requiring a second code always introduce immortal time into any study utilizing the algorithm. Suissa defines immortal time as “a span of cohort follow-up during which, because of exposure definition, the outcome under study could not occur.” [4] Studies that inadvertently or deliberately require immortal time prior to the outcome have been long criticized. These studies commonly begin follow-up after the initiation of treatment, which cannot be confirmed until subsequent events occur after initiation (e.g., a second prescription, an additional therapy or procedure, etc.). Immortal time occurs when the subjects who die, disenroll from the database, or experience the outcome before the second requirement can be satisfied are not included in the analysis. Agarwal et al. discuss the immortal time incurred between a mastectomy and radiation therapy on patient survival [5]. The time between the surgery and the radiation treatment is considered immortal and must be accounted for in the treatment effect analysis.

Studies that require a second diagnosis code in a phenotype algorithm incur similar immortal time. These studies pose the potential for incorporating immortal time bias into analyses in cases where the data window used to determine inclusion in the cohort overlaps the data window used to assess outcome incidence. When using these algorithms, non-fatal outcomes of interest may occur between the time from the first diagnosis code to the second diagnosis code, as the first code is considered the cohort index date. The principal concern using these algorithms is the occurrence of death, which prevents a second code as required by the algorithm. The bias in this case is due to the possible loss of sicker subjects from the cohort. While these studies are similar to other forms of immortal time incurred in observational research, the effect of requiring a second diagnosis code has not been well studied.

A trade-off exists between the need to increase the likelihood of a research subject having the condition of interest, i.e., increasing specificity, when using the known errors in administrative claims data and the incorporation of immortal time. The objectives of this study were to 1) estimate the performance characteristics of phenotype algorithms that require a second code to increase specificity; 2) to estimate the immortal time associated with each algorithm; and 3) quantify the impact on incidence rate estimates for various outcomes across various time-at-risk definitions.

## Materials and methods

We examined algorithms for the following 11 chronic health conditions: atrial fibrillation, chronic kidney disease, chronic heart failure, coronary artery disease, migraine, multiple myeloma, multiple sclerosis, overactive bladder, plaque psoriasis, psoriatic arthritis, and ulcerative colitis. In this study, these 11 conditions are used as exposures or indications (i.e., the index event that represents entry into the study and the beginning of follow-up time). The analyses were from data from five databases: Merative^®^ MarketScan^®^ Commercial Database (CCAE), Multi-State Medicaid Database (MDCD), and Medicare Supplemental Database (MDCR), Optum^®^'s Clinformatics^®^ Data Mart (DOD), and IQVIA^®^ Adjudicated Health Plan Claims Data (formerly PharMetrics Plus)—US database (PharMetrics). The results presented in this study are the mean values from the databases used in the analysis. For each health condition, we created five algorithms to examine performance [sensitivity and positive predictive value (PPV)] differences. We created a broad algorithm with a single code for the health condition, which incorporates no immortal time and would potentially have high sensitivity and low specificity. We also created four narrow algorithms with potentially higher specificity (and lower sensitivity) where a second diagnosis code was required at one of four time periods after the first diagnosis code: 1–30 days, 1–90 days, 1–365 days, and 1- all days in a subject’s observed data after the first code. In these cohorts, subjects may have the outcome prior to the occurrence of the second code. For these algorithm definitions, the principal concern with immortal time is death or disenrollment rather than missed outcome occurrence. As disenrollment is likely to be health status agnostic, we will focus on death as the main effect of immortal time.

For each algorithm requiring a second code, the time between cohort entry (the first diagnosis) and the required second diagnosis is immortal. We examined the proportion of immortal time relative to time-at-risk (TAR) for four outcomes: death, myocardial infarction, Bell’s palsy, and ingrown toenails. For these analyses, we only included data from DOD and MDCD, as these were more reliable for accurately recording death. For each outcome, we used four possible TARs for each chronic health condition: 0–30 days after the first condition occurrence (the index date), 0–90 days post-index, 0–365 days post-index, and 0–1,095 days post-index. Performance characteristics of algorithms for chronic health conditions were estimated using PheValuator (V2.1.4) from the Observational Health Data Sciences and Informatics (OHDSI) toolstack (for a complete description of this tool).^1^ This method uses diagnostic predictive modeling to estimate the probability of subjects being cases of the condition of interest. It provides the complete set of performance characteristics, i.e., sensitivity, specificity, and positive and negative predictive value. Using the semi-automated phenotype algorithm evaluation method PheValuator, we eliminated the need for obtaining and reviewing subject’s records. While algorithm validation results from chart review are considered the “gold standard,” we have compared the results from PheValuator with prior studies using chart review and found excellent agreement between the two methods [6]. Immortal time was calculated as the time from the index date until the first of the following: 1) the outcome; 2) the end of the outcome TAR; or 3) the occurrence of the second code for the chronic health condition. Total TAR was calculated as the total time across subjects from the index date until the end of TAR. We calculated the proportion of immortal time relative to the total TAR as total immortal time/total TAR. Incidence rates were calculated as the count of the first occurrence of the event for each subject during the TAR divided by the total TAR for all subjects. We used a lookback period (“clean window”) of a minimum of 365 days to designate an incident event.

All phenotype algorithms used in this study were developed using the OHDSI open-source ATLAS tool.^2^ Each algorithm is available in a JSON format.^3^ The JSON format may be converted into executable SQL using either the ATLAS or CirceR^4^ packages.

The preliminary results from this study were originally presented at the Observational Health Data Sciences and Informatics (OHDSI) symposium in 2022 [7].

## Results

**FIGURE 1:**
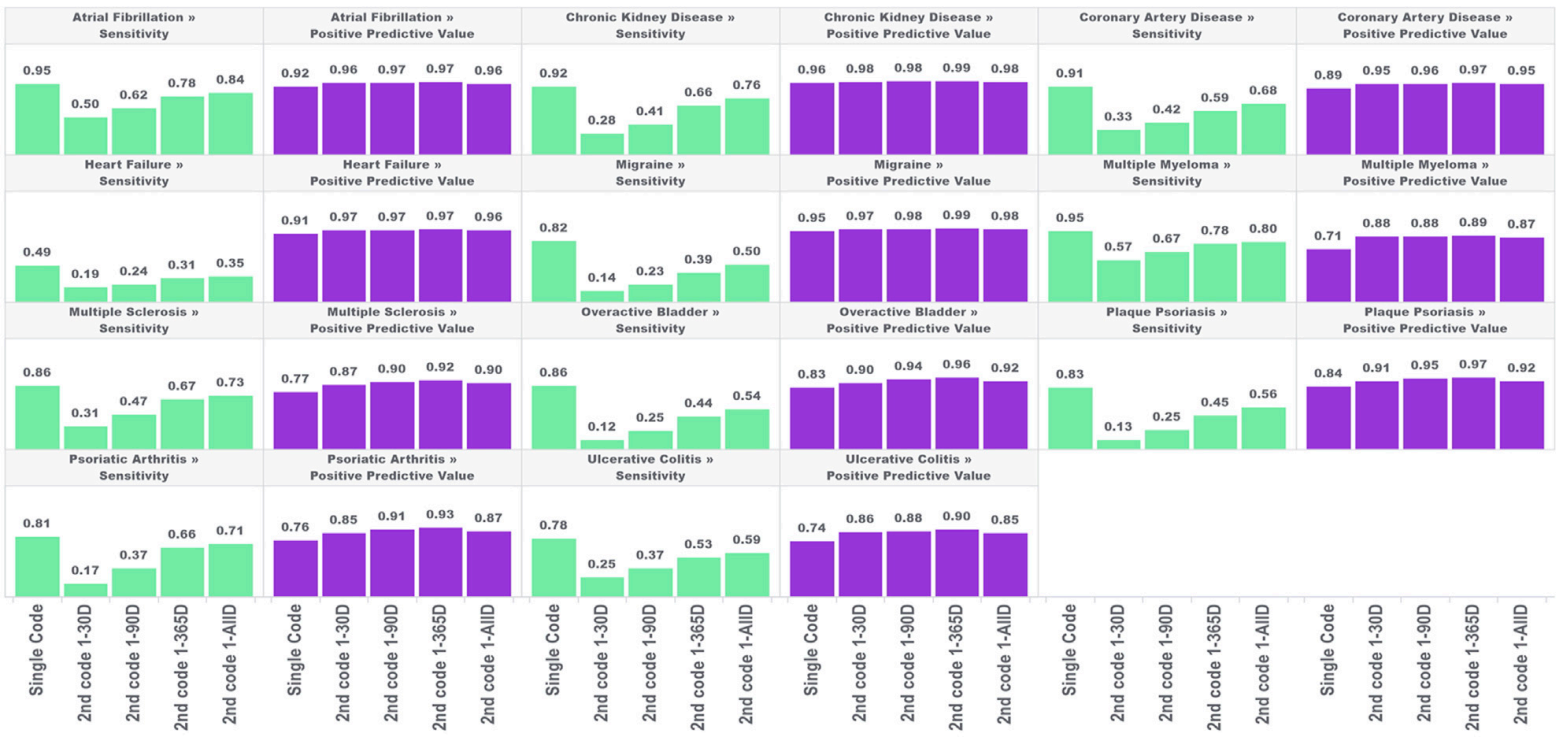
Performance characteristics of 11 chronic conditions by phenotype algorithm aggregated across five databases using PheValuator.

In the first analysis, we estimated the sensitivity and PPV for the single-code algorithm and the four algorithms requiring a second condition code for each of the 11 chronic conditions across five databases. The results are shown in Figure 1.

In each of these conditions, the narrow phenotype algorithms, i.e., those requiring a second condition code, produced higher estimates for PPV and lower estimates for sensitivity compared to the single code algorithm. For all conditions studied, PPV was highest in the algorithm requiring a second code 1–365 days after index, with moderate decreases in PPV when the second code was allowed to occur any other time after the first. Adding a second code had more of an impact on PPV in some conditions than in others. For example, adding the requirement of a second code 1–365 days after the index for multiple myeloma produced a 25% increase in PPV (0.71, single code, 0.89, two codes). For chronic kidney disease, adding a second code produced a 3% increase in PPV (0.96, single code, 0.99, two codes). The differences in PPV with any of the four versions of the two-coded algorithms were generally small. The largest difference was observed in psoriatic arthritis, where requiring a second code 1–30 days after index produced an estimated PPV of 0.85 which increased to 0.93 with the 1–365 days algorithm (∼9%).

The improvements in PPV came at the cost of decreased sensitivity. In all conditions, increasing the time to the required second code increased the sensitivity of the algorithm. However, in all conditions, the sensitivity of the two-code algorithms was lower than that of the single-code algorithms, regardless of the required time for the second code. There was substantial variation in the decrease in sensitivity depending on the time required for the second code and the condition type. For conditions with more serious and immediate clinical consequences, there was a smaller drop in sensitivity from the single code algorithm compared to the two-code algorithms for conditions with less critical immediate clinical consequences. For example, for atrial fibrillation, the sensitivity decreased from 0.95 to 0.50 when requiring a code 1–30 days after index (−47%), while for overactive bladder the sensitivity decreased from 0.82 to 0.14 (−83%). For the algorithms requiring a second code at 1-all days after index, atrial fibrillation sensitivity decreased from 0.95 to 0.84 (−9%); for overactive bladder sensitivity decreased from 0.82 to 0.50 (−39%).

**FIGURE 2:**
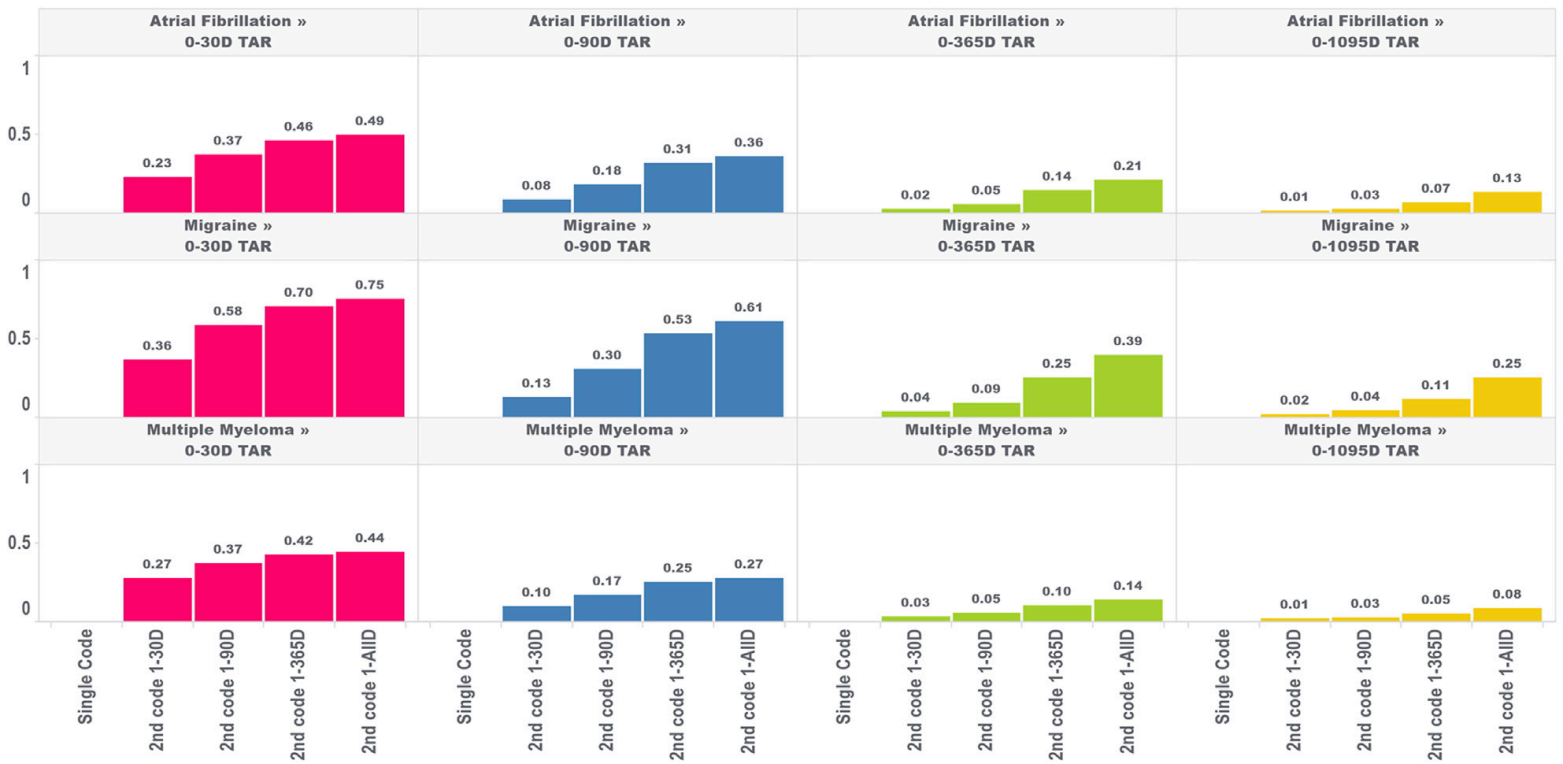
Proportion of immortal time relative to total TAR for atrial fibrillation, migraine, and multiple myeloma with an outcome of death for multiple outcome times-at-risk (TAR) by phenotype algorithm aggregated across five databases using PheValuator.

In the second analysis, we calculated the proportion of TAR that would be immortal (i.e., time where subjects cannot die or leave the database) for four outcomes and four TARs, with the results for the outcome of death shown in Figure 2.

The amount of immortal time in the analysis increased as the window used to identify the second diagnosis code increased. For example, in a 30 days TAR incidence rate analysis conducted on cohorts where the second diagnosis code was allowed any time after the first (i.e., 1-ALL), 49%, 75%, and 44% of TAR was immortal for the atrial fibrillation, migraine, and multiple myeloma cohorts, respectively, compared to 23%, 36%, and 27% when the second code was required to occur within 30 days of the first. Furthermore, the proportion of TAR that was immortal was highest when the maximum-allowable TAR in the incidence rate analysis was short and decreased as the maximum-allowable TAR increased. The proportion of immortal time was substantially lower for the 1,095 days TAR analyses. For migraine cohorts requiring 2 codes, which had the highest proportions of immortal time among our four outcomes, we found the 1,095 days TAR analysis had 25% of TAR immortal for cohorts requiring the second code any time after the first code and 2% of TAR immortal for cohorts requiring the second code within 30 days of the first code. We found similar results for this analysis in the other three outcomes.

We found different trends for changes in incidence rates for death and ingrown toenail in chronic diseases, depending on the time to second occurrence of condition codes and the outcome TAR. For all target cohorts and both outcomes, we observed higher incidence rate estimates when a second code was required within 30 days of the original, which opposes the direction of the bias expected due to immortal person time (which deflates incidence rates). This increase most likely reflects genuinely higher incidence rates of death or ingrown toenail among subjects who have two related codes within 30 days of each other. This may be due to subjects who have more frequent encounters with the health system being at higher risk of death and also being more likely to have outcomes captured in the data.

**FIGURE 3:**
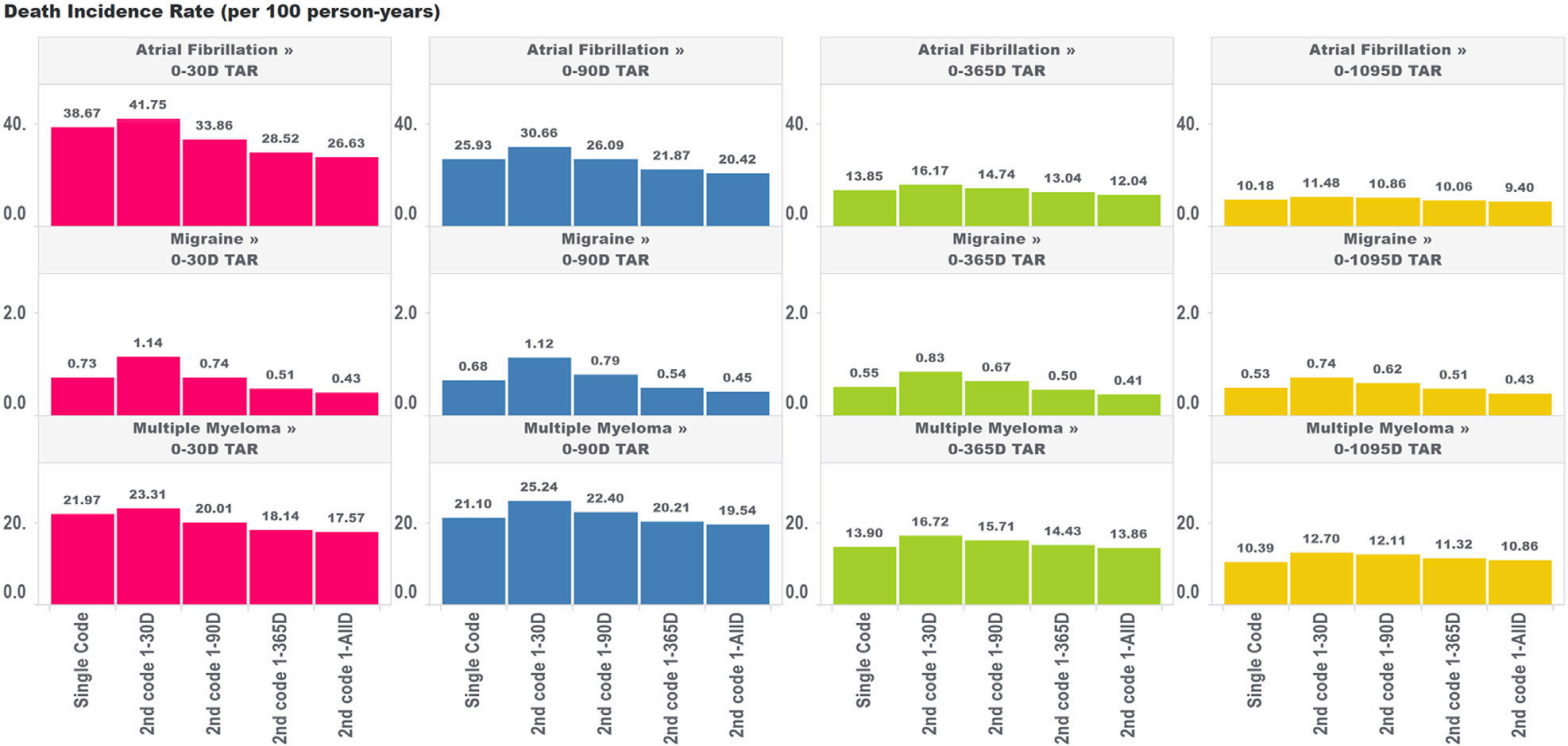
Effect of requiring a second code in phenotype algorithms for atrial fibrillation, migraine, and multiple myeloma on incidence rates of death for multiple outcome times-at-risk (TAR) by phenotype algorithm aggregated across five databases using PheValuator.

For the mortality analysis (Figure 3), as we extended the time period used to allow for the second code, we observed two phenomena working in the same direction: 1) a reduction in the incidence rate of death due to including subjects who are less intensely medicalized (who didn’t have two diagnosis codes in very close proximity to each other) and 2) a false reduction in the incidence rate due to immortal time representing an increasing share of the total TAR. It is impossible to entirely parse these two drivers of change in estimates; however, we can observe that the variation in estimates across algorithms requiring the second code at different intervals was largest for the incidence rate analyses that included the largest proportion of TAR that was immortal.

**FIGURE 4:**
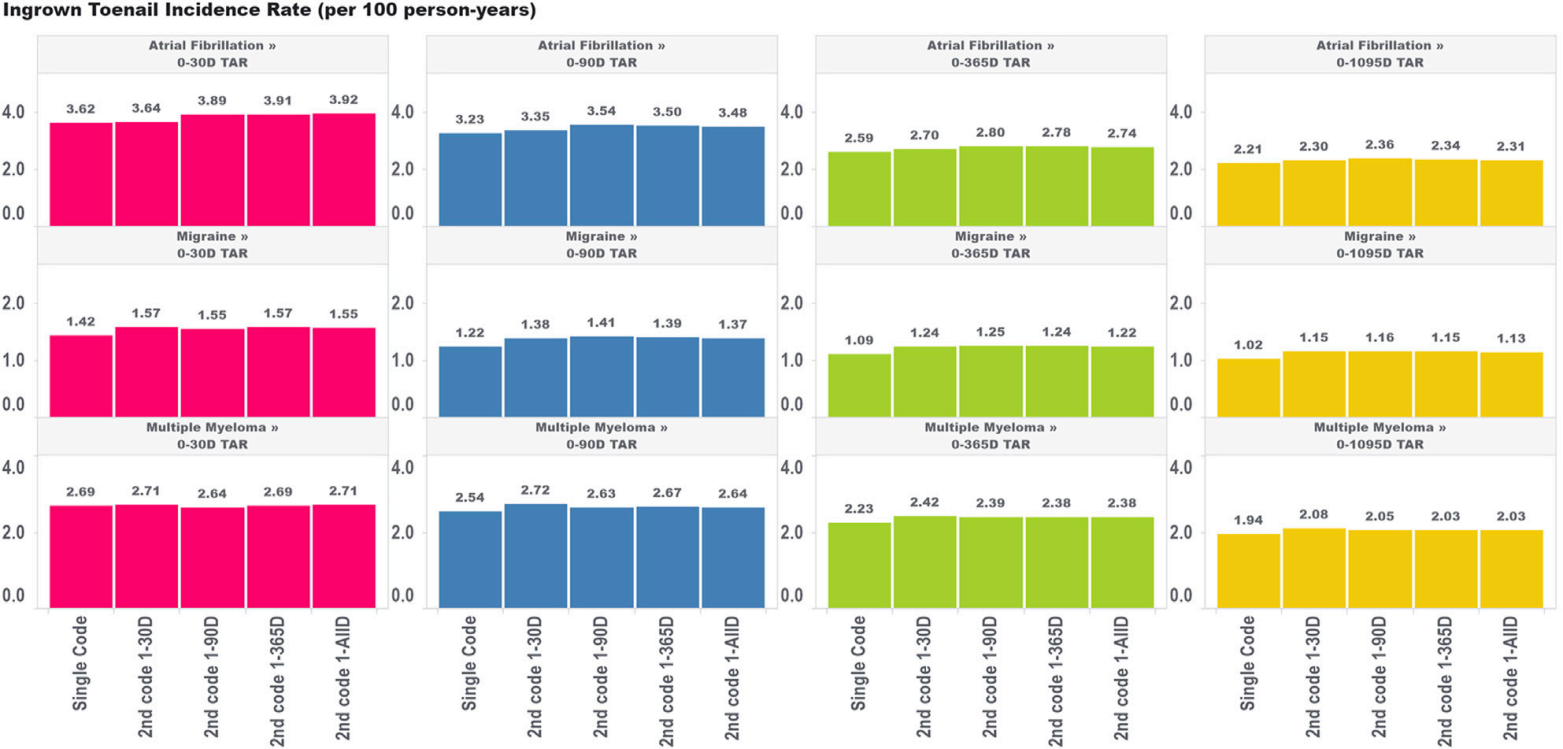
Effect of requiring a second code in phenotype algorithms for atrial fibrillation, migraine, and multiple myeloma on incidence rates of ingrown toenail for multiple outcome times-at-risk (TAR) by phenotype algorithm aggregated across five databases using PheValuator.

For ingrown toenail (Figure 4), an outcome that we expected to be much less susceptible to immortal time bias due to its limited association with mortality, we saw much less variation in the incidence rate estimates across the various algorithms requiring a second code within different time windows. This provides some evidence that immortal time is not meaningfully biasing these estimates. Indeed, we still saw an increase in the incidence rate comparing the single-code algorithm to the algorithm requiring a second code within 30 days, which indicates that the aforementioned effects of having frequent encounters with the health system are still affecting incidence rate estimates to some degree for ingrown toenails (particularly for the migraine cohorts). However, extending the period used to observe the second code, which we found increased immortal time in the analysis, did not appear to have a meaningful impact on incidence rate estimates. This provides some insight that immortal time should only be of concern for high-mortality cohorts where outcomes are meaningfully associated with the risk of mortality.

## Discussion

In this study, we examined the effect of creating narrow phenotype algorithms by varying the time to require a second condition code in the algorithm compared to broad algorithms, i.e., those requiring a single code. We found that requiring a second code at any time after the index event (i.e., the occurrence of a condition code for the first time in a subject’s history) always increased the positive predictive value and decreased the sensitivity compared to single-code algorithms. Requiring a second code 1–365 days post-index produced the highest estimates for positive predictive value. Requiring a second code 1–30 days post-index produced the lowest estimates for sensitivity. In the 11 health conditions we examined, the trade-off of positive predictive value gain to sensitivity loss was lower for certain conditions, particularly those where the single-code algorithm had a high positive predictive value. We also found that requiring a second code invariably adds immortal time to the subjects in these cohorts. The proportion of immortal time relative to the outcome time-at-risk was highest for short outcome times-at-risk and lower for long times-at-risk. Attempting to increase the specificity of a health condition algorithm by adding a second code is a potentially valid approach for the five US databases we tested. However, caution should be observed in analyses where the outcome is death or outcomes highly associated with death due to the necessary incorporation of immortal time, especially in analyses assessing short periods of follow-up.

Bias due to immortal time for incidence rate estimates is a complex function depending on several factors, including the distribution of TAR in the analysis, the background risk of mortality in the target population, the association of the outcome with mortality, and the shape of the hazard function over follow-up [4, 8]. The impact of using various algorithms requiring a second code on incidence rates of death and ingrown toenails is shown in Figures 3, 4, respectively. *A priori*, we expected that the ingrown toenail outcome would likely show limited bias due to immortal time as there should be almost no increased risk of death among people diagnosed with ingrown toenails. Researchers should always consider applying quantitative bias analysis (QBA) to understand the degree of bias incurred using any phenotype algorithm in a study. Many authors have provided guidelines for assessing bias due to immortal time [9, 10]. Liang et al. provide a useful guide for performing QBA specifically to inform bias incurred by immortal time for non-fatal outcomes [11]. The work of Lash et al. provides a useful guide for QBA in general [12]. PheValuator is a useful tool to determine the performance characteristics of phenotype algorithms to be used in QBA calculations.

There are several strengths in the present study. First, this study developed phenotypes using data from five large datasets that reflect subjects of a wide range of ages and from various socioeconomic backgrounds. The approach we used for the development of the phenotypes in this study uses publicly available, open-source software, providing the capability for full result replication. Included in the supplemental information are the JSON files, which provide fully reproducible phenotype algorithms. There were also several limitations to our study, which included the use of administrative datasets primarily maintained for insurance billing that are well-known to have significant deficits, including coding inaccuracies [13]. In addition, the estimation of performance characteristics using the PheValuator methodology is dependent on the quality of the data in the dataset, which can vary substantially [14]. Incomplete signs and symptoms documentation in the data could affect the accuracy of the index date. The algorithm validation was performed using a method, PheValuator, involving predictive modeling rather than case review. This method does have the advantage of providing performance characteristics for multiple databases. It also provides the full set of performance metrics, including sensitivity and specificity which are rarely provided in validation studies using case reviews [15]. The results from PheValuator have also been compared to the results from previously published validation studies and have demonstrated excellent agreement [6]. In the algorithms defined with only one diagnostic code, it was not possible to determine if any of these were rule-out diagnoses. Lastly, our study observed data from those subjects who presented for medical attention; those who did not seek medical attention but had the disease were not included and may affect the metrics in this study. Those with less severe disease may also not have sought medical attention.

## Conclusion

The work presented here provides evidence for the cost and benefits of using narrow and broad phenotype algorithms in epidemiological research. The benefits of a narrow phenotype are shown in higher positive predictive values for the health condition of interest, assuring researchers that they are studying the correct condition. The cost comes from incurring immortal time into the study design. We have shown this to be a significant issue when examining severe outcomes such as death but of minor impact when examining outcomes with low mortality rates. Researchers with study designs where a narrow phenotype algorithm is needed might consider alternative approaches to using a second code, such as requiring a specific procedure or other treatment within a very short period following the diagnosis code.

## Data Availability

The data analyzed in this study is subject to the following licenses/restrictions: The license to use the data sets were purchased from Optum, Merative, and IQVIA. The identical datasets may be purchased by other researchers. Requests to access these datasets should be directed to Merative Marketscan: marketscan.support@merative.com; Optum: maria.restuccio@optum.com; IQVIA: www.iqvia.com.

## Footnotes

1 See: https://github.com/OHDSI/PheValuator

2
https://atlas.ohdsi.org/#/home

3
https://github.com/OHDSI/PheValuator/tree/develop/extras/ImmortalTimeBias

4
https://github.com/OHDSI/CirceR
